# On the General Dedekind Sums and Two-Term Exponential Sums

**DOI:** 10.1155/2014/146926

**Published:** 2014-10-14

**Authors:** Junli Zhang, Wenpeng Zhang

**Affiliations:** ^1^Xi'an Eurasia University, Xi'an 710065, China; ^2^School of Mathematics, Northwest University, Xi'an 710127, China

## Abstract

We use the analytic methods and the properties of Gauss sums to study the computational problem of one kind hybrid mean value involving the general Dedekind sums and the two-term exponential sums, and give an interesting computational formula for it.

## 1. Introduction

Let *q* be a natural number and *h* an integer prime to *q*. The classical Dedekind sums

(1) $$\begin{array}{c} S\left(h,q\right)=\overset{q}{\underset{a=1}{\sum }}\left(\left(\frac{a}{q}\right)\right)\left(\left(\frac{ah}{q}\right)\right), \end{array}$$

where

(2) $$\begin{array}{c} \left(\left(x\right)\right)=\{\begin{array}{cc} x-\left[x\right]-\frac{1}{2}, & \text{if}\;x\;\text{is}\;\text{not}\;\text{an}\;\text{integer};\\ 0, & \text{if}\;x\;\text{is}\;\text{an}\;\text{integer}, \end{array} \end{array}$$

describes the behaviour of the logarithm of the eta-function (see [1, 2]) under modular transformations. About the various arithmetical properties of *S*(*h*, *q*), many people had studied it and obtained a series of interesting results; see [3–9].

For example, Wang and Zhang [6] and Wang and Pan [7] had studied the hybrid mean value involving Dedekind sums and two-term exponential sums and proved the computational formulae

(3) ∑m=1p−1 ∑n=1p−1|C(m,n,3,1;p)|2·S(mn¯,p)  ={p·hp2,if  p=12k+7,3p·hp2,if  p=12k+11,0,if  p=4k+1,∑m=1p−1 ∑n=1p−1|C(m,n,4,2;p)|2·S(mn¯,p)  ={2p·hp2,if  p=8k+7,0,if  p=4k+1  or  8k+3,

where *h*_*p*_ denotes the class number of the quadratic field $Q\left(\sqrt{-p}\right)$, $\bar{n}$ denotes the solution of the congruence equation *nx* ≡ 1mod⁡ *p*, and the two-term exponential sums *C*(*m*, *n*, *h*, *k*; *q*) are defined as

(4) $$\begin{array}{c} C\left(m,n,h,k;q\right)=\overset{q}{\underset{a=1}{\sum }}e\left(\frac{ma^{h}+na^{k}}{q}\right), \end{array}$$

*e*(*y*) = *e* ^2*πiy*^. Some results related to *C*(*m*, *n*, *h*, *k*; *q*) can be found in [10, 11].

On the other hand, Zhang [12] introduced a generalized Dedekind sums as follows:

(5) $$\begin{array}{c} S\left(h,n;q\right)=\overset{q}{\underset{a=1}{\sum }}{\bar{B}}_{n}\left(\frac{a}{q}\right){\bar{B}}_{n}\left(\frac{ah}{q}\right), \end{array}$$

where

(6) $$\begin{array}{c} {\bar{B}}_{n}\left(x\right)=\{\begin{array}{cc} B_{n}\left(x-\left[x\right]\right), & \text{if}\;x\;\text{is}\;\text{not}\;\text{an}\;\text{integer};\\ 0, & \text{if}\;x\;\text{is}\;\text{an}\;\text{integer}, \end{array} \end{array}$$

*B*_*n*_(*x*) denotes the *n*th Bernoulli polynomial and ${\bar{B}}_{n}(x)$ defined for all real 0 < *x* ≤ 1 is called the *n*th Bernoulli periodic function.

If *n* = 1, then *S*(*h*, 1; *q*) = *S*(*h*, *q*), the classical Dedekind sums. About the arithmetical properties of *S*(*h*, *n*; *q*) and *B*_*n*_(*x*), one can find them in [3, 12]. In this paper as a note of [6, 7], we consider the following hybrid mean value:

(7) $$\begin{array}{c} \overset{p-1}{\underset{u=1}{\sum }}\;\overset{p-1}{\underset{v=1}{\sum }}C\left(m,u,h,k;p\right)\cdot C\left(m,v,h,k;p\right)\cdot S\left(u\cdot \bar{v},n;p\right) \end{array}$$

and use the analytic methods and the properties of Gauss sums to give an exact computational formula for (7). That is, we will prove the following conclusion.

**Theorem 1 .**

Let *p* ≥ 3 be a prime and *n* any positive integer. Then for any positive integers *h* and *k* with (*hk*, *p* − 1) = 1 and integer *m* with (*m*, *p*) = 1, one has the identity

(8) ∑u=1p−1 ∑v=1p−1C(u,m,h,k;p)·C(v,m,h,k;p)·S(u·v¯,n;p)  =p2·S(1,n;p).

For *n* = 1, 2, and 3, from our theorem we may immediately deduce the following.

**Corollary 2 .**

Let *p* ≥ 3 be a prime. Then for any positive integers *h* and *k* with (*hk*, *p* − 1) = 1 and integer *m* with (*m*, *p*) = 1, one has the identity

(9) ∑u=1p−1 ∑v=1p−1C(u,m,h,k;p)·C(v,m,h,k;p)·S(u·v¯,1;p)  =112·p(p−1)(p−2).

**Corollary 3 .**

Let *p* ≥ 3 be a prime. Then for any positive integers *h* and *k* with (*hk*, *p* − 1) = 1 and integer *m* with (*m*, *p*) = 1, one has the identity

(10) ∑u=1p−1 ∑v=1p−1C(u,m,h,k;p)·C(v,m,h,k;p)·S(u·v¯,2;p)  =1180·(p−1)(p3−4p2+6p+6)p.

**Corollary 4 .**

Let *p* ≥ 3 be a prime. Then for any positive integers *h* and *k* with (*hk*, *p* − 1) = 1 and integer *m* with (*m*, *p*) = 1, one has the identity

(11) ∑u=1p−1 ∑v=1p−1C(u,m,h,k;p)·C(v,m,h,k;p)·S(u·v¯,3;p)  =1840·(p−2)(p−1)(p+1)(p+2)(p2+5)p3.

For general integer *q* > 3, whether there exists an exact computational formula for the hybrid mean value

(12) $$\begin{array}{c} \overset{q}{\underset{u=1}{\sum }}\;{\;}^{\prime }\overset{q}{\underset{v=1}{\sum }}\;{\;}^{\prime }C\left(u,m,h,k;q\right)\cdot C\left(v,m,h,k;q\right)\cdot S\left(u\cdot \bar{v},n;q\right) \end{array}$$

is an open problem, where *h* and *k* are positive integers with (*kh*, *ϕ*(*q*)) = 1 and (*m*, *q*) = 1.

## 2. Several Lemmas

In this section, we will give two lemmas, which are necessary in the proof of our theorem. Hereinafter, we will use many properties of character sums and Gauss sums; all of these can be found in [13], so they will not be repeated here. First we have the following.

**Lemma 1 .**

Let *p* be an odd prime and *χ* any nonprincipal character mod⁡  *p*. Then for any positive integers *h* and *k* ≥ 1 with (*hk*, *p* − 1) = 1 and any integer *m*, one has the identity

(13) $$\begin{array}{c} \overset{p-1}{\underset{u=1}{\sum }}\chi \left(u\right)\cdot C\left(u,m,h,k;p\right)=\chi^{\bar{k}\cdot h}\left(m\right)\cdot \tau \left(\chi \right)\cdot \tau \left({\bar{\chi }}^{\bar{k}\cdot h}\right), \end{array}$$

where *τ*(*χ*) = ∑_*a*=1_^*p*−1^ *χ*(*a*) *e* (*a*/*p*) denotes the Gauss sums.

**Proof**

From the definitions of *C*(*u*, *m*, *h*, *k*; *p*) and Gauss sums we have

(14) ∑u=1p−1χ(u)·C(u,m,h,k;p)  =∑u=1p−1χ(u)∑a=1pe(uah+makp)  =∑a=1p ∑u=1p−1χ(u)·e(uah+makp)  =τ(χ)∑a=1p−1χ¯h(a)·e(makp).

Since (*hk*, *p* − 1) = 1, then there exits one integer $\bar{k}$ such that $\bar{k}\cdot k\equiv 1\mathrm{mod}\;(p-1)$ and $(\bar{k},p-1)=1$. From the properties of reduced residue system mod⁡ *p* we know that if *a* pass through a reduced residue system mod⁡ *p*, then $a^{\bar{k}}$ also pass through a reduced residue system mod⁡ *p*. So from (14) and Fermat little theorem we have

(15) ∑u=1p−1χ(u)·C(u,m,h,k;p)  =τ(χ)∑a=1p−1χ¯h(ak¯)·e(mak¯·kp)  =τ(χ)∑a=1p−1χ¯k¯·h(a)·e(map)  =χk¯·h(m)·τ(χ)·τ(χ¯k¯·h).

This proves Lemma 1.

**Lemma 2 .**

Let *q* ≥ 3 be an integer and *h* any integer with (*h*, *q*) = 1. Then for any positive integer *n*, one has the following identities.

(i) If *n* is an odd number, then
  (16) $$\begin{array}{c} S\left(h,n;q\right)=\frac{{\left(n!\right)}^{2}}{4^{n-1}q^{2n-1}\pi^{2n}}\underset{d\;|\;q}{\sum }\frac{d^{2n}}{\phi \left(d\right)}\underset{\chi \left(-1\right)=-1}{\underset{\chi \mathrm{mod}\;d}{\sum }}\chi \left(h\right){\left|L\left(n,\chi \right)\right|}^{2}. \end{array}$$
(ii) If *n* is an even number, then
  (17) S(h,n;q)=(n!)24n−1q2n−1π2n∑d ∣ qd2nϕ(d) ×∑χmod⁡ dχ(−1)=1χ(h)|L(n,χ)|2−(n!)24n−1π2n·ζ2(n),

where *L*(1, *χ*) denotes the Dirichlet *L*-function corresponding to character *χ*mod⁡ *d* and *ζ*(*s*) is the famous Riemann zeta-function.

**Proof**

See [12].

## 3. Proof of the Theorem

In this section, we will complete the proof of our theorem. If *n* is an odd number, then note that for any nonprincipal character *χ*mod⁡ *p*, $|\tau (\chi )|=\sqrt{p}$ and $\tau (\chi )\cdot \tau \left(\bar{\chi }\right)=\bar{\chi }(-1)\tau (\chi )\bar{\tau (\chi )}=\bar{\chi }(-1)\cdot p$. So for any integer *u* with (*u*, *p*) = 1, from (i) of Lemma 2 we have

(18) ∑u=1p−1∑v=1p−1C(u,m,h,k;p)·C(v,m,h,k;p)·S(u·v¯,n;p)  =(n!)2·p4n−1(p−1)π2n   ×∑χmod⁡ pχ(−1)=−1∑u=1p−1∑v=1p−1C(u,m,h,k;p)  ·C(v,m,h,k;p)χ(u·v¯)|L(n,χ)|2  =(n!)2·p4n−1(p−1)π2n∑χmod⁡ pχ(−1)=−1χ¯(−1)χk¯·h(−1)·p2  ·|L(n,χ)|2  =(n!)2·p34n−1(p−1)π2n∑χmod⁡ pχ(−1)=−1|L(n,χ)|2=p2·S(1,n;p).

If *n* ≥ 2 is an even number, then note that (*k*, *p* − 1) = 1 and the identity

(19) ∑u=1p−1C(u,m,h,k;p)  =∑a=1p ∑u=1p−1e(uah+makp)  =p−1+∑a=1p−1e(makp)∑u=1p−1e(uahp)  =p−1−∑a=1p−1e(map)=p.

From (ii) of Lemma 2 and the method of proving (18) we have

(20) ∑u=1p−1 ‍ ∑v=1p−1C(u,m,h,k;p)·C(v,m,h,k;p)·S(u·v¯,n;p)  =(n!)2·p4n−1(p−1)π2n   ×∑χmod⁡ p χ(−1)=1χ≠χ0∑u=1p−1 ∑v=1p−1C(u,m,h,k;p)  ·C(v,m,h,k;p)χ(u·v¯)|L(n,χ)|2   +(n!)2·p24n−1p2n−1π2n·ζ2(n)+(n!)2·p4n−1(p−1)π2n   ·p2·|L(n,χ0)|2−(n!)2·p24n−1π2nζ2(n)  =(n!)2·p34n−1(p−1)π2n∑χmod⁡ pχ(−1)=1|L(n,χ)|2   +(n!)2·p24n−1p2n−1π2n·ζ2(n)−(n!)2·p24n−1π2nζ2(n)  =(n!)2·p24n−1p2n−1π2n∑d ∣ pd2nϕ(d)∑χmod⁡ dχ(−1)=1|L(n,χ)|2   −(n!)2·p24n−1π2n·ζ2(n)  =p2·S(1,n;p).

Combining (18) and (20) we may immediately complete the proof of our theorem.

From the definition of *S*(1,1; *p*) we have

(21) S(1,1;p)=∑a=1p−1(ap−12)2=112(p−3+2p)=(p−1)(p−2)12p.

Combining (18) and (21) we can deduce the identity

(22) ∑u=1p−1 ∑v=1p−1C(u,m,h,k;p)·C(v,m,h,k;p)·S(u·v¯,1;p)  =112·p(p−1)(p−2).

This proves Corollary 2.

If *n* = 2, then note that *B*_2_(*x*) = *x* ^2^ − *x* + 1/6; we have

(23) S(1,2;q)=∑a=1q−1B¯22(aq)=∑a=1q−1(a2q2−aq+16)2=q180−136+118q−130q3.

From (20) and (23) we have the identity

(24) ∑u=1p−1 ∑v=1p−1C(u,m,h,k;p)·C(v,m,h,k;p)·S(u·v¯,2;p)  =1180·(p−1)(p3−4p2+6p+6)p.

This proves Corollary 3.

If *n* = 3, then note that *B*_3_(*x*) = *x* ^3^ − (3/2)*x* ^2^ + (1/2)*x*; we have

(25) S(1,3;q)=∑a=1q−1B¯32(aq)=∑a=1q−1(a3q3−32a2q2+12aq)2=q840−140q3+142q5.

From (18) and (23) we have the identity

(26) ∑u=1p−1∑v=1p−1C(u,m,h,k;p)·C(v,m,h,k;p)·S(u·v¯,3;p)  =1840·(p−2)(p−1)(p+1)(p+2)(p2+5)p3.

This completes the proof of Corollary 4.
